# Lack of replication for the myosin-18B association with mathematical ability in independent cohorts

**DOI:** 10.1111/gbb.12213

**Published:** 2015-04-01

**Authors:** K A Pettigrew, S F Fajutrao Valles, K Moll, K Northstone, S Ring, C Pennell, C Wang, R Leavett, M E Hayiou-Thomas, P Thompson, N H Simpson, S E Fisher, A J O Whitehouse, M J Snowling, D F Newbury, S Paracchini

**Affiliations:** † School of Medicine, University of St AndrewsSt Andrews, UK; ‡ Department of Child and Adolescent Psychiatry, Psychosomatics, and Psychotherapy, Ludwig-Maximilians-UniversityMunich, Germany; § Department of Psychology, University of York; ¶ School of Social and Community Medicine, University of BristolUK; ** School of Women's and Infants' Health, University of Western AustraliaCrawley, Australia; †† Department of Experimental Psychology; ‡‡ Wellcome Trust Centre for Human Genetics, Oxford UniversityUK; §§ Max Planck Institute for Psycholinguistics; ¶¶ Donders Institute for Brain, Cognition and Behaviour, Radboud UniversityNijmegen, The Netherlands; *** Telethon Kids Institute, University of Western Australia (M560)Crawley, Australia; ††† St. Johns College, University of OxfordUK; ‡‡‡ A list of SLIC members can be found in the Acknowledgements

**Keywords:** ALSPAC, cognitive abilities, dyscalculia, dyslexia, genetic association, neurodevelopmental disorders

## Abstract

Twin studies indicate that dyscalculia (or mathematical disability) is caused partly by a genetic component, which is yet to be understood at the molecular level. Recently, a coding variant (rs133885) in the myosin-18B gene was shown to be associated with mathematical abilities with a specific effect among children with dyslexia. This association represents one of the most significant genetic associations reported to date for mathematical abilities and the only one reaching genome-wide statistical significance. We conducted a replication study in different cohorts to assess the effect of rs133885 maths-related measures. The study was conducted primarily using the Avon Longitudinal Study of Parents and Children (ALSPAC), (*N* = 3819). We tested additional cohorts including the York Cohort, the Specific Language Impairment Consortium (SLIC) cohort and the Raine Cohort, and stratified them for a definition of dyslexia whenever possible. We did not observe any associations between rs133885 in myosin-18B and mathematical abilities among individuals with dyslexia or in the general population. Our results suggest that the myosin-18B variant is unlikely to be a main factor contributing to mathematical abilities.

Mathematical ability is a skill essential for an individual's academic and employment outcomes as well as everyday activities. Dyscalculia is a condition where mathematical ability is severely impaired and is recognized as a clinical syndrome in the World Health Organisation (WHO) International Classification of Diseases (Mental and Behavioural Disorders, ICD-10) and Diagnostic and Statistical Manual of Mental Disorders (DSM-5). Individuals with dyscalculia have profound difficulties in acquiring basic mathematical skills, in the absence of a general cognitive impairment and despite access to adequate educational opportunities (Butterworth *et al.* 2011). Dyscalculia is characterized by high heterogeneity of symptoms and impairment on a range of basic computational skills, such as counting, number fact knowledge, written calculation and mathematical reasoning. Dyscalculia usually presents early in childhood (Kaufmann & Von Aster 2012), with a prevalence estimate of 3–6% (Devine *et al.* 2013). These deficits persist into adulthood and cause major challenges for academic performance and occupational opportunities, particularly if untreated.

Similar to other neurodevelopmental disorders, such as dyslexia, attention deficit hyperactivity disorders (ADHD) or specific language impairment (SLI), dyscalculia has a clear neurobiological and genetic basis (Butterworth & Kovas 2013). Twin studies have demonstrated that mathematical ability is a trait determined, at least partly, by genetic factors with estimated heritability for low mathematical performance of 0.65 (Haworth *et al.* 2009) and 0.69 (Oliver *et al.* 2004). Dyscalculia presents significant comorbidity with other neurodevelopmental disorders such as dyslexia (Landerl & Moll 2010; Moll *et al.* 2014b), ADHD (Czamara *et al.* 2013) and SLI (Donlan *et al.* 2007).

Dyscalculia can be considered as the lower tail of the phenotypic distribution of mathematical abilities across the general population. Hypothesizing a shared biological component, it is possible that the same genetic factors contributing to mathematical abilities are also implicated in dyscalculia. Such hypothesis is supported by what is observed for dyslexia, a specific impairment in learning to read (Habib & Giraud 2013). It has been shown that some dyslexia candidate genes also influence reading abilities in the general population, both including as well as excluding individuals who met a definition for dyslexia (Paracchini 2011). For example, the *KIAA0319* gene, originally identified in independent cohorts selected for dyslexia (Newbury *et al.* 2014), was associated with single word reading and single word spelling in the Avon Study of Parents and Children (ALSPAC), a longitudinal cohort representing the general population (Paracchini *et al.* 2008; Scerri *et al.* 2011).

Molecular genetic studies for dyscalculia have been sparse and, so far, a limited number of genetic variants have been proposed to influence mathematical abilities. Two genome-wide association studies (GWASs) for mathematical abilities did not report any significant associations (Baron-Cohen *et al.* 2014; Docherty *et al.* 2010). A third GWAS confirmed a significant genetic component underlying mathematical abilities, but did not identify specific risk factors (Davis *et al.* 2014).

The rs133885 variant in the myosin-18B (*MYO18B*) gene is the only marker that has been found to be associated with mathematical ability at statistically significant level, as reported in a separate study (Ludwig *et al.* 2013). The association was identified in an initial discovery sample of 200 individuals diagnosed with dyslexia and then replicated in two other dyslexia cohorts of German or Austrian origin (*N* = 699 total, effect size 4.87%, *P* = 7.71 × 10^−10^). The association and relatively large effect size appear to be specific in dyslexia cohorts. The same variant showed a weaker, but still statistically significant, association in a general population cohort from the UK (Twin Early Development study or TEDS) (*N* = 1080, effect size 0.26%, *P* = 0.048) and the same trend was observed in a combined general population sample (*N* = 1471, effect size 0.007%, *P* = 0.075). rs133885 is a missense variant and was therefore indicated to be directly causative. Neuroimaging of 79 healthy adults showed that carriers of the rs133885 risk genotype, associated with low mathematical performance, displayed a reduced depth of the right intraparietal sulcus. Numerical processing has long been understood to be localized to the parietal lobes (Nieder & Dehaene 2009) but more recent studies suggest that this occurs more specifically in the intraparietal sulcus (Bugden *et al.* 2012). The *MYO18B* association is possibly the most robust association with mathematical abilities reported so far; however, it has not been independently replicated. Replication of this association is particularly challenging as it requires the availability of large cohorts characterized with a wide range of cognitive tests including both mathematical and reading measures.

We conducted the first replication analysis of the genetic association between rs133885 and mathematical ability in several cohorts: the Avon Longitudinal Study of Parents and Children (ALSPAC) (*N* = 3819), the York Cohort (*N*_total_ = 291), the Specific Language Impairment Consortium (SLIC) (*N*_total_ = 367) and the Raine cohort (*N* = 667), for a total of *N* = 5144 individuals. These cohorts were stratified for a dyslexia definition where possible and relevant. We found no evidence of association and our data suggest rs133885 is not a major and common factor contributing to maths skills.

## Materials and methods

### Samples

#### The ALSPAC cohort

ALSPAC is a longitudinal cohort representing the general population living in the Bristol area. The ALSPAC cohort consists of over 15 000 children from the southwest of England that had expected dates of delivery between 1 April 1991 and 31 December 1992. From age 7, all children were invited annually for assessments on a wide range of physical, behavioural and neuropsychological traits, including cognitive (reading and mathematics related) measures. DNA is available for approximately 11 000 ALSPAC children. Informed written consent was obtained from the parents after receiving a complete description of the study at the time of enrolment into the ALSPAC project, with the option for them or their children to withdraw at any time. Ethical approval for the present study was obtained from the ALSPAC Law and Ethics Committee and the Local Research Ethics Committees (Boyd *et al.* 2013).

We used two maths scores; (1) the arithmetic subtest of the Wechsler Intelligence Scale for Children (WISC) (Wechsler 1992), which consists of verbal maths problems that require basic calculation skills and (2) a maths achievement factor score (MA; Nunes *et al.* 2012), derived from UK national curriculum maths tests taken between 10 and 14 years of age (Table1). The variables were selected because they predominantly focused on basic computational skills, similar to those used in the TEDS study (Ludwig *et al.* 2013). Data on these measures approximate a normal distribution.

**Table 1:** Description of cohorts analysed for genetic association

Cohort	Cohort type	*N*, Overall cohort	*N*, Dyslexia subgroup	Phenotypes	Comparable phenotypes in discovery and replication samples (Ludwig *et al.* 2013)
**ALSPAC**	Epidemiological Longitudinal Singleton	3819	329	WISC: arithmetic – verbal word problems with time limit (Wechsler 1992)	TEDS: using and applying mathematics number tasks (e.g. counting) (iii) perception of shapes, space and measures
				MA (Nunes *et al.* 2012)	
**York**	Clinical Longitudinal Family	109 families (291 total individuals)	72 families	NJ (dot counting and number transcoding) (Moll *et al.* 2014a, 2014b)	NJ (object counting and number transcoding) in Munich sample
				(201 total individuals; including language impairment status)		
				MC (calculation efficiency: addition and multiplication) in Munich sample, Austrian sample and German/Austrian control sample	MC (calculation efficiency: addition and subtraction) (Moll *et al.* 2014a)
				WIAT-NO (calculation accuracy) (Wechsler 2005)	
				GMF	
**SLIC**	Clinical Epidemiological Family	169 families (367 total individuals)		WISC-III: arithmetic – verbal word problems with time limit (Wechsler 1992)	
				WAIS-III: arithmetic – verbal word problems with time limit (Wechsler 1997)	
**RAINE**	Epidemiological Longitudinal Singleton	667		MA (WALNA-numeracy: written word problems) (Western Australian Government Department of Education and Training 2012)	TEDS: using and applying mathematics number tasks (e.g. counting) perception of shapes, space and measures

WAIS, Wechsler Adult Intelligence Scale.

**Figure 1:**
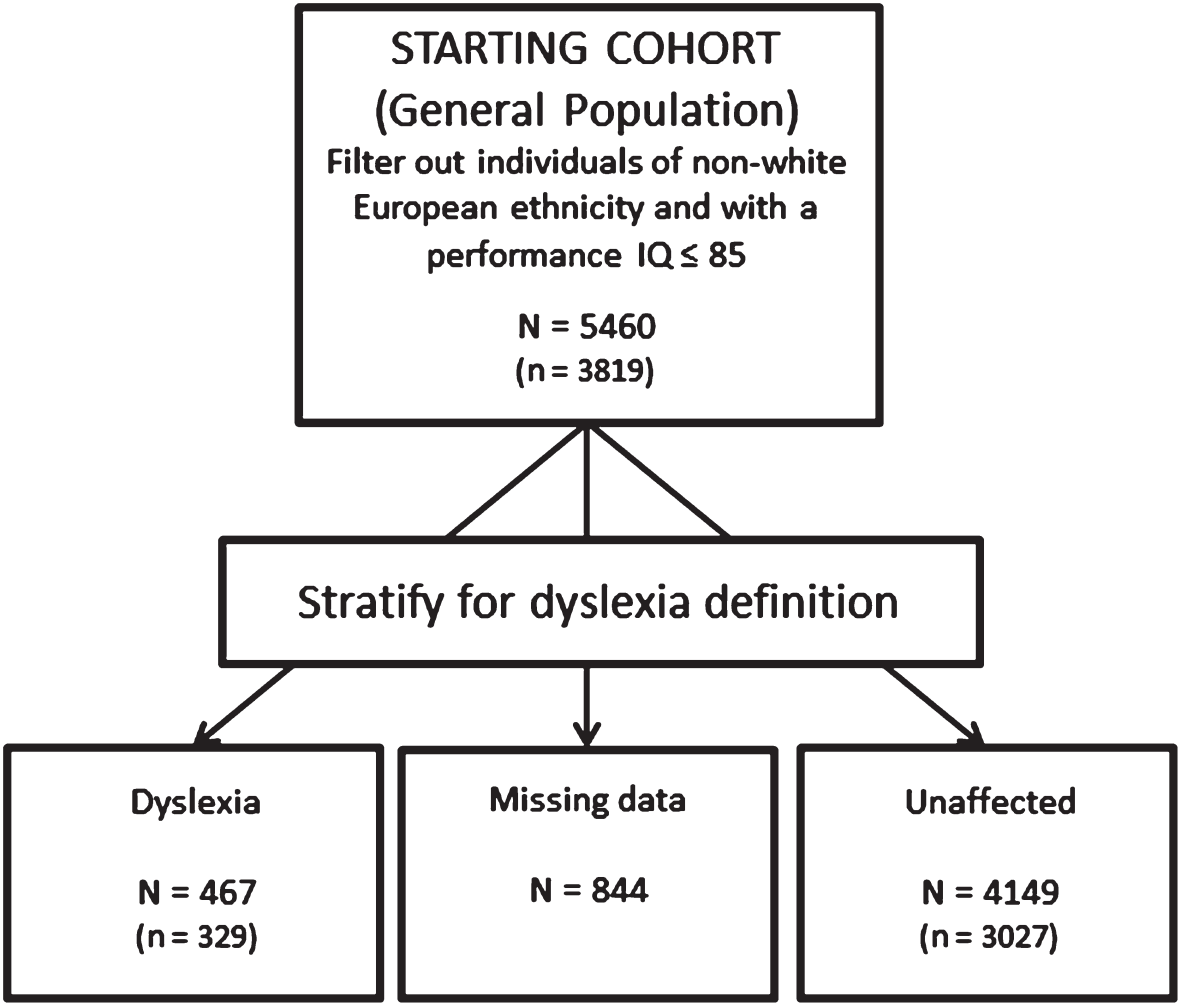
Definition of ALSPAC children cohort samples used for analysis. An initial subgroup of *N* = 5460 was identified after filtering out individual of non-White European origin and with a performance IQ ≤ 85. Within this subgroup we stratified the sample upon a definition of dyslexia. Numbers of individuals included in the association analysis for having a complete set of genotypes and phenotypes are in brackets.

We included ALSPAC participants with white European ethnicity to avoid confounding effects of population stratification, and with a performance IQ > 85 to avoid the possibility that low reading and maths performance were related to a general cognitive impairment, similarly to our previous analysis in the same cohort (Scerri *et al.* 2011). These criteria led to a sample of 5460 individuals (Fig. 1). From this group participants were considered to have dyslexia if they scored < −1 standard deviation (SD) for single word reading at both 7 and 9 years of age. In total, *N* = 467 individuals met criteria for dyslexia, while *N* = 4149 were assigned to the unaffected subgroup. The remaining individuals had incomplete reading data, and were excluded from subgroup analysis (*N* = 844).

The ALSPAC study website contains details of all the data that is available through a fully searchable data dictionary http://www.bris.ac.uk/alspac/researchers/data-access/data-dictionary/.

#### The York cohort

The York cohort is a longitudinal cohort designed to study the development of reading and language difficulties in young children (Nash *et al.* 2013). It includes 116 families for a total of 304 individuals. Families of probands with a performance IQ less than 85, or of non-white European origin, were excluded from the analysis. The analysis was run in the complete filtered dataset (*N* = 109 families; *N* = 291 individuals) as well as in a subgroup of families with a history of dyslexia and/or having a child with language impairment (impaired subgroup; *N* = 72 families; *N* = 201 individuals). The individual phenotypes selected for association analysis were: (1) numerosity judgement (NJ) derived from two tests, dot counting and number transcoding (NT, composite of number recoding and writing), (2) mathematical calculation (MC) (Moll *et al.* 2014a) based on timed addition and subtraction tests and on the numerical operation subtest of the Wechsler Individual Achievement Test (WIAT-NO; Wechsler 2005; Table1). NJ and MC (i.e. the timed addition and subtraction subtests) are analogous to the constructs investigated in the original study. Principal components analysis indicated that all four variables contributed to a single global maths factor (GMF), in accordance with a previous report (Schulte-Korne *et al.* 2007). This GMF was used as an additional phenotype for association analysis (Table1). Correlation between these measures showed a *R*^2^ value ranging from 0.4 and 0.7 (Table2).

**Table 2:** Correlations coefficients (*r*) between maths scores in cohorts in which participants underwent multiple tests

		WISC	MA	NT	NJ	MC	WIAT-NO	GMF
**ALSPAC**	**WISC**	1						
	**MA**	0.5036	1					
**York**	**NT**			1				
	**NJ**			0.5480	1			
	**MC**			0.5408	0.4992	1		
	**WIAT-NO**			0.5285	0.4507	0.7231	1	
	**GMF**			0.7983	0.7600	0.8629	0.8304	1

The study was approved by NHS Research Ethics (Yorkshire & The Humber Bridge – Humber Bridge) and the University of York Department of Psychology Ethics Committee.

#### The SLIC cohort

The SLIC cohort is a family-based cohort collected to study language impairment. This cohort has been described previously in detail (Falcaro *et al.* 2008; Specific Language Impairment Consortium (SLIC) 2002; Specific Language Impairment Consortium (SLIC) 2004). Briefly, these nuclear families were collected from five sites around the UK (Guys Hospital, London, Cambridge, Manchester, Edinburgh and Aberdeen). All selected families had a single proband showing language skills ≥ 1.5 SD below the mean for their age and nonverbal IQ scores within the specified normal range (>80). DNA was collected from all immediate family members regardless of language status. Ethical approval was given by local ethics committees. A subsample was extracted for the current study on the basis of families for whom data was available for the arithmetic subtest of the WISC-III (Wechsler 1992) (verbal maths problems as score (1) in the ALSPAC sample) or the Wechsler Adult Intelligence Test (WAIS-III) (Wechsler 1997) as appropriate. In total, the subsample consisted of 681 individuals from 169 nuclear two-generation families and included 367 individuals with phenotype data (308 children and 59 adults) and 605 individuals with genotype data for rs133885.

#### The Raine cohort

The Western Australian Pregnancy Cohort (Raine) Study was started as a randomized controlled trial to evaluate the effects of repeated ultrasound in pregnant women in Perth, Western Australia. In total, 2900 pregnant women were recruited between 1989 and 1991 prior to 18 weeks gestation at the King Edward Memorial Hospital (Perth, Western Australia) (Newnham *et al.* 1993). Women were randomized to repeated ultrasound measurements at 18, 24, 28, 34 and 38 weeks gestation or to a single ultrasound assessment at 18 weeks. Children have been assessed at average ages of 1, 2, 3, 5, 8, 10, 14 and 17 and both height and weight were collected at each assessment. The study was conducted with appropriate institutional ethics approval (ethics approval number for DNA collection and storage: EC03-14.7 and EC06-29), and written informed consent was obtained from mothers at all follow-ups and participants at the year 17 follow-up. Included individuals (*N* = 667) had (1) no known intellectual or neurosensory disability, (2) a nonverbal IQ score ≥ 4th percentile on the Raven's Colored Progressive Matrices, corresponding to approximately > −1.75 SD the population average of the 50th centile, and (3) biological parents who were both of white European origin. The phenotype used was the numeracy outcome variable, which is similar to the mathematical achievement (MA) score used in ALSPAC, and is derived from The Western Australian Literacy and Numeracy Assessment (WALNA) (Western Australian Government Department of Education and Training 2012) (Table1). The WALNA is composed of word problems, testing a range of constructs which include maths reasoning, geometry and calculation.

### Genotyping and statistical analysis

Genotype data for rs133885 in the ALSPAC, SLIC and Raine cohorts were extracted from genome-wide genotyping dataset previously generated and filtered following standard quality control procedures (Anderson *et al.* 2010; Nudel *et al.* 2014). The York cohort was genotyped using a TaqMan assay (LifeTechologies, Paisley, UK). All cohorts, including a significant subsets of the York cohort for which genome-wide genotype data were available, have been checked for population stratification in previous analyses. The few outliers were removed before we conducted the analysis. Quantitative association analysis was conducted using plink (Purcell *et al.* 2007) in unrelated individuals and QTDT for families (Abecasis *et al.* 2000), modelling for an additive effect unless otherwise specified. Power calculations were conducted using the Genetic Power Calculator (Purcell *et al.* 2003).

## Results

**Figure 2:**
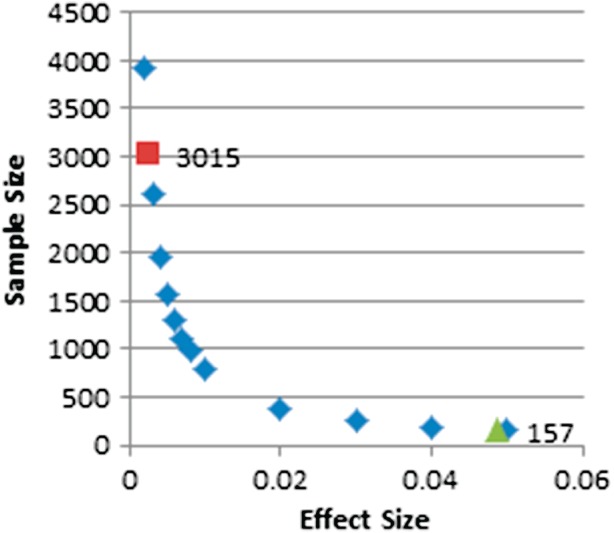
Power calculations. The graph shows the sample sizes required to detect different effect sizes as predicted by power calculations assuming a minor allele frequency of 0.45 and with α = 0.05. The green triangle and the red square indicate that samples of 157 and 3015 have > 80% power to detect an effect size of 4.87% and 0.26% respectively.

We assessed whether our samples had sufficient genetic power to find genuine associations between rs133885 and mathematical abilities based on the study that originally reported this association (Ludwig *et al.* 2013; Fig. 2).

Ludwig and colleagues reported different effect sizes in the subgroups they analysed on the basis of a dyslexia definition. We assumed an effect size of 4.87% which was reported in the combined sample of individuals with dyslexia (*N* = 699) and an effect size of 0.26% in a general population sample (*N* = 1080). We assumed the variant was directly functional, as suggested in the original study, and we modelled the calculation for a singleton cohort, an allele frequency of 0.45 based on our general population cohort (ALSPAC) with α = 0.05 (Fig. 2). The observed allele frequency is very similar to what is reported for a European population (MAF = 46%) by the HapMap project (http://hapmap.ncbi.nlm.nih.gov/index.html.en). The analysis predicted that sample sizes of 157 and 3015 were required to achieve > 80% power to detect an effect size of 4.87% and 0.26% respectively. The ALSPAC subgroups (Table1), which are our primary sample for investigation, were therefore predicted to have sufficient power to detect the previously reported association between rs133885 and mathematical abilities in the general population cohort (*N* = 3819), as well as in the unaffected group (*N* = 3027), following stratification for dyslexia, with an effect size of 0.26%. The dyslexia subgroup (*N* = 329) had more than 80% power to detect an effect size of 4.87%, which however is a very large effect on the basis of what we would expect for complex traits. The smallest effect size our general population sample (*N* = 3819) was predicted to detect was 0.21% (>80% power) and 0.275% (>90% power). The smallest effect sizes the dyslexia subgroup (*N* = 329) could detect was 2.4% (>80% power) and 3.2% (>90% power).

We conducted an association analysis selecting available phenotypes that would best match those used in the original report (Table1). The analysis in the discovery sample used a ‘basic mathematical ability factor’, combining scores of ‘mathematical calculation’ and ‘numerosity judgement” (Ludwig *et al.* 2013), which was not available in ALSPAC. The arithmetic (WISC) and the MA phenotypes used in the ALSPAC cohort were comparable to the phenotypes of the TEDS sample which was used as replication cohort and was representative of the general population. These two maths scores had a correlation of *r* = 0.5036 (Table2). We ran the association analysis using both an additive and a genotypic model. We could not detect any association signal, either in the general population cohort (WISC, *N* = 4302, *P* = 0.8571, β = −0.004; MA, *N* = 3819, *P* = 0.3206, β = −0.023), the unaffected general population (WISC, *N* = 3378, *P* = 0.7798, β = 0.007; MA, *N* = 3027, *P* = 0.3091, β = −0.025) or in the dyslexia subgroup (WISC, *N* = 369, *P* = 0.6172, β = −0.033; MA, *N* = 329, *P* = 0.6444, β = 0.034). The statistics above refer to results obtained under an additive model which yielded relatively smaller *P*-values compared to a genotypic model.

To further investigate this association we then extended the analysis to additional cohorts for which both mathematical and reading measures were available. The York cohort has been characterized with a large number of cognitive tests including mathematical measures. The cohort has been primarily collected to study the development of language and reading development in young children with a family history of dyslexia and/or exhibiting a language deficit. We analysed both the entire cohort (*N* = 109 families, *N* = 291 individuals) and a subgroup of families selected for the proband having language difficulties or a family history of dyslexia, as a single group to avoid analyzing very small sample sets (*N* = 74 families, *N* = 201 individuals). We tested a wider range of maths-related phenotypes (Table1) and we did not detect any association (minimum *P*-value = 0.1312, NJ, impaired subgroup). Under the same assumptions reported in Fig. 2, and therefore modelling for *N* = 109 unrelated singletons, the minimum effect size that could be detected in this small cohort (>80% power) is 7%.

The SLIC cohort was recruited on the basis of a language impairment diagnosis and, as predicted by the comorbidity across SLI and dyslexia, many children in this cohort present reading difficulties. We therefore did not split this cohort according to the presence or absence of dyslexia. Of the 348 individuals with mathematics and reading/spelling data (59 parents and 289 children), 21% had reading or spelling ability greater than 1.5 SD below than that expected for their age. Of the children alone, 22.8% had reading or spelling abilities more than 1.5SD below that expected for their age. No association (minimum *P*-value = 0.8836, WISC-III arithmetic) was found for rs133885 and the available maths measures (Table1). Under the assumptions shown in Fig. 2, the minimum effect size that could be detected in this cohort is 4.75%. This is smaller than the effect size reported in the dyslexia cohort by Ludwig et al. (2013).

The Raine cohort is an epidemiological longitudinal cohort representing the general population. The cohort was filtered for ethnicity and to remove individuals presenting sensory or neurological problems that would have impacted their maths scores for specific reasons. We did not filter the cohort for a dyslexia definition, because that would have led to a sample size too small to be analysed. We ran association analysis in 667 individuals for a MA score (Table1) and detected no association (*P*-value = 0.737). Under the assumptions shown in Fig. 2, the minimum effect size that could be detected in this cohort is 1.18%. This is larger than the effect size reported for the general population sample by Ludwig et al. (2013) (0.26%).

## Discussion

We have conducted the first independent replication study for the previously reported associations between rs133885 in the myosin-18B gene and mathematical abilities identified through a GWAS (Ludwig *et al.* 2013). We used several independent cohorts including ALSPAC (*N* = 3819 individuals), the York cohort (*N* = 109 families, *N* = 291 individuals) and the SLIC (*N* = 169 families, *N* = 367 individuals) and Raine cohorts (*N* = 667 individuals). Consistently with the original study, we conducted our analysis stratifying the samples for a dyslexia definition, when possible. We could not detect any association between rs133885 and maths abilities.

Power calculations predicted that sample sizes of 157 and 3015 were required to replicate the original finding in a dyslexia and general population cohort, respectively. The sample size in the ALSPAC cohort exceeded these numbers. One difference in our analysis stems from the different phenotypes used for quantitative association analysis (Table1). The phenotype data collected in the ALSPAC study was selected to mirror as closely as possible the tests used in the original investigation (Ludwig *et al.* 2013), but the measures available in ALSPAC were largely restricted to UK National Curriculum maths examination results. These are designed to test a range of mathematical abilities, including basic calculation skills, word problems, number concept and perception of shape, space and time. The measures we used in ALSPAC (arithmetic subtest of the WISC and MA score, Table1) are sufficiently comparable to the phenotypes used in the UK-based TEDS general population replication cohort as reported by Ludwig and colleagues (Ludwig *et al.* 2013). We would have expected to see an effect in the ALSPAC general population and unaffected cohorts (*N* = 3819 and *N* = 3027 respectively) which were approximately 3 times the size of TEDS (*N* = 1080). Therefore, our analysis does not support the role of this variant in contributing significantly to maths abilities. It is possible that the lack of replication can be attributed to an over estimation of the effect size in the discovery sample according to the well-established phenomenon known as ‘winner's curse’ (Zollner & Pritchard 2007). This could be very well the case in the light of the large effect size reported for the *MYO18B* variant of 4.87% in the combined sample of individuals (*N* = 699) with dyslexia. This unusually large effect size and strength of association compared with what is generally observed for other complex traits is driven by the discovery sample (*N* = 200) where the reported effect size was of 15.78%. This is an extremely large effect size especially for a common marker (rs133885 MAF = 45%) and was most likely an overestimation of any potential genuine associations. The associations observed in the replication samples showed consistent trends of associations but with weaker strengths and effect sizes, only marginally contributing to the global association. Given the small sample size, it is possible the association in the discovery sample was a false positive, driving the signal of the combined dataset.

In addition to the ALSPAC samples we investigated other cohorts, smaller in size and therefore underpowered to detect small effects, but which allowed further exploration of any possible trend of association. The York cohort is smaller in size but has been characterized extensively for numerical skills and is enriched for children presenting language and reading difficulties. In particular, the maths phenotypes of the York cohort are in line with those used by Ludwig et al. (Ludwig *et al.* 2013) presenting both components of the combined measure used in the original study: mathematical calculation (MC, i.e. timed arithmetic skills) and numerosity judgement (NJ, i.e. counting) abilities. We analysed both the whole cohort, which included typically developing children, and a subset selected on the basis of reading and language impairment. We extended our subgroup to include language impaired children because of the extensive comorbidity between reading and language disorders, and to avoid running the analysis in a very small sample. On this point it is worth mentioning that the original finding (Ludwig *et al.* 2013) detected association in the dyslexia subgroup because dyslexia was the phenotype of interest. The phenotypes available in the dyslexia cohort of the original study were different from those used in the replication samples. Therefore, it is possible that the strength of the *MYO18B* association in the discovery sample is specific to the phenotype used, rather than to a dyslexia definition. Ethnicity is another factor that could explain lack of replicability. The original study (Ludwig *et al.* 2013) included cohorts of individuals with dyslexia with German or Austrian origin. This factor may not simply indicate an ethnic-specific effect but may underlie differences in dyslexia definition and in ascertainment criteria for study participants. In Germany, a dyslexia diagnosis is based mainly on reading fluency and spelling abilities, while in the UK it relies mainly on reading accuracy. Therefore, even if we stratified for a dyslexia definition, we might have selected a different population subset in which the rs133885 effect is not detectable. The SLIC (*N* = 367) and Raine (*N* = 667) cohorts are larger than the York cohort but less well-characterized with mathematical measures making direct comparisons more challenging because of inconsistency across available phenotypes. The high variability of measures described here also highlights a particular challenge for genetic investigations of cognitive traits. Our analysis demonstrates how difficult it is to make direct comparisons across different studies collected and assessed under variable criteria. Establishing universal or more directly comparable strategies, which will make it possible to match different studies and ideally to combine samples, would be an important advance for the field of cognitive and neurodevelopmental trait genetics.

The field of complex trait genetics, as other research areas, is becoming increasingly aware of publication bias towards positive findings (Munafo 2009) and the importance of reporting negative replications. Therefore our study, conducted in an adequately powered sample, contributes to a balanced interpretation of the significance of genetic findings.

In this study, we were unable to replicate the association between rs133885 in the myosin-18B gene and mathematical abilities. Although we could not reconstruct the exact study design, we based our analysis on a large sample and extended our investigations to several independent cohorts. We conclude that the *MYO18B* variant is not contributing to mathematical skills in general.
